# Gold nanoplatform for near-infrared light-activated radio-photothermal gas therapy in breast cancer

**DOI:** 10.3389/fbioe.2022.1098986

**Published:** 2023-01-06

**Authors:** Shuting Zuo, Zhenyu Wang, Liping Zhao, Jing Wang

**Affiliations:** ^1^ Department of Breast Surgery, The Second Hospital of Jilin University, Changchun, China; ^2^ Gynecology and Obstetrics Department of the Second Hospital of Jilin University, Changchun, China

**Keywords:** nitric oxide, gas therapy, radio-sensitization, combination therapies, breast cancer

## Abstract

Although radiotherapy is one of the most common treatments for triple-negative breast cancer (TNBC), it frequently has unsatisfactory therapeutic outcomes due to the radiation resistance of tumor tissues. Therefore, a synergistic strategy is urgently needed to increase therapeutic responses and prolong patient survival. Herein, we constructed gold nanocages (GNCs) loaded with a hyperpyrexia-sensitive nitric oxide (NO) donor (thiolate cupferron) to integrate extrinsic radiosensitization, local photothermal therapy, and near-infrared-activated NO gas therapy. The resulting nanoplatform (GNCs@NO) showed a high photothermal conversion efficiency, which induced the death of cancer cells and facilitated rapid NO release in tumor tissues. The radiosensitizing efficacy of GNCs@NO was further demonstrated *in vitro* and *in vivo*. Importantly, the released NO reacted with the reactive oxide species induced by radiotherapy to produce more toxic reactive nitrogen species, exerting a synergistic effect to improve anticancer efficacy. Thus, GNCs@NO demonstrated excellent effects as a combination therapy with few adverse effects. Our work proposes a promising nanoplatform for the radio/photothermal/gas treatment of TNBC.

## 1 Introduction

Triple-negative breast cancer (TNBC) is an extremely malignant tumor that threatens the lives of women worldwide (Lyons, 2019; Cortes et al., 2022). Although radiotherapy and chemotherapy after surgical resection are the standard treatments for TNBC, they often result in unsatisfactory outcomes due to radio/chemoresistance and severe side effects (James et al., 2019; Baranova et al., 2022). Thus, highly effective and safe treatment methods for TNBC are urgently needed to prolong patient survival and improve their quality of life.

Gas therapy has attracted considerable attention owing to its non-invasiveness and absence of drug tolerance (Dai et al., 2018; Freund and Bekeschus, 2020). More importantly, gas therapy often exerts synergistic effects with other treatment modalities, including radiotherapy, chemotherapy, and photodynamic therapy, because of the regulation of cancer cells and tumor microenvironment (Fan et al., 2018; Liu et al., 2020; Wu et al., 2021). For example, carbon monoxide (CO) gas can effectively decrease tumor resistance to chemotherapy or radiotherapy by inhibiting cytochrome c oxidase and cytochrome P450 enzymes, causing obstacles in respiratory chain transmission and difficulties in oxygen utilization (Giuffrè et al., 2020; Thakar et al., 2021). Nitric oxide (NO) is the first gasotransmitter in the gas family and has various physiological and pathological activities, including blood vessel relaxation, NO poisoning, and macrophage activation (Yu et al., 2018; Ding et al., 2019). Furthermore, NO can react with reactive oxygen species (ROS) to produce more lethal reactive nitrogen species (RNS), which improves the effectiveness of radiotherapy as the treatment effect of radiotherapy is mainly due to ROS produced by the interaction between radiation and water (Zuo et al., 2022). Therefore, combining radiotherapy with NO therapy promises a synergistic effect with low side effects for the treatment of TNBC. However, it remains a challenge to deliver unstable NO to the target site for maximal efficacy.

Photothermal therapy (PTT) is a promising green treatment because of its non-invasiveness (Fernandes et al., 2020; Gao et al., 2021). PTT alleviates hypoxia in tumor tissues by increasing tumor blood perfusion, which may improve the radiosensitivity of tumor tissues (Wang et al., 2019). Gold nanocages (GNCs) with tunable optical properties matching the near-infrared region (NIR) are particularly attractive for PTT because of their excellent photothermal conversion effect (Wang et al., 2018; Alimardani et al., 2021). Furthermore, GNCs, as high-Z nanomaterials, have emerged as radiosensitizers owing to their enhanced radiation absorption (Chen et al., 2020; Penninckx et al., 2020). More importantly, GNCs are widely used as nanodrug delivery systems because of their unique porous walls, hollow structure, and surface that can be easily functionalized (Wang et al., 2018; Zhang et al., 2021). Therefore, GNCs are ideal nanocarriers for delivering NO donors to provide radiosensitization, PTT, and gas therapy for treatment with spatiotemporal consistency.

**SCHEME 1:**
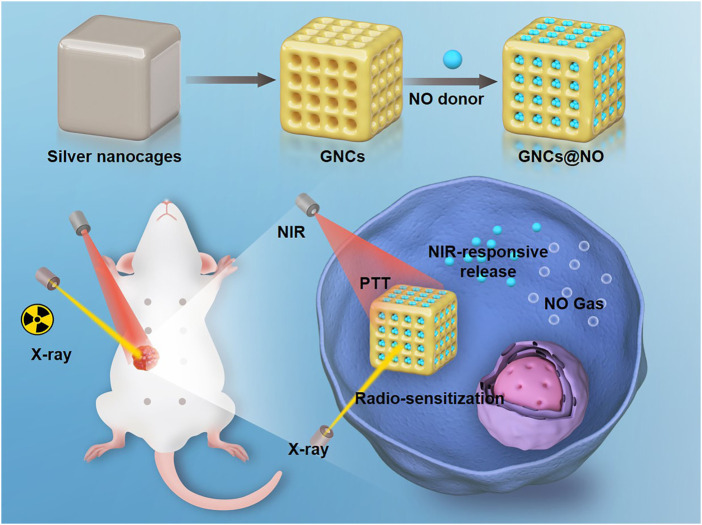
Fabrication of nitric oxide (NO) donor-loaded gold nanocages for radiosensitization, local photothermal therapy, and NIR-activated NO gas therapy for breast cancer.

In this work, we prepared GNCs and preloaded them with a hyperpyrexia-sensitive NO donor, thiolate cupferron, through thiol–gold interactions to form GNCs@NO (Scheme 1). GNCs@NO showed a high loading ability of the NO donor, good photothermal conversion efficiency, and NIR-stimulated NO release. *In vitro* and *in vivo* experiments demonstrated multiple therapeutic effects, including PTT and NO poisoning, as well as radiosensitization. More importantly, the released NO showed a synergistic effect with radiotherapy through the production of RNS by reacting with ROS. Thus, the combination of GNCs@NO and X-rays induced a remarkable antitumor effect and low systemic toxicity. Overall, our work proposes a promising nanoplatform for the multimodal treatment of TNBC.

## 2 Methods

### 2.1 GNC synthesis

GNCs were fabricated using a galvanic replacement reaction between HAuCl_4_ and silver nanocages as previously described (Tang et al., 2021). First, we prepared 5 ml of 0.2 mg/ml aqueous polyvinyl pyrrolidone (PVP). Subsequently, we added silver nanocages (5 mg) to the prepared PVP solution. The mixture was then heated at 100°C for 30 min. Subsequently, 1 mM HAuCl_4_ was slowly added to the mixture and reacted for another 30 min. Finally, the solution was cooled to room temperature and centrifuged at 8000 rpm for 15 min to collect GNCs. The GNCs were repeatedly washed with ethyl alcohol and distilled water before storage at 4°C.

### 2.2 NO donor loading and NO release

First, 10 mg thiolate cupferron was dissolved in dimethyl sulfoxide (DMSO) (0.5 ml). Subsequently, the thiolate cupferron solution was mixed with 20 ml of 0.1 nM aqueous GNCs and stirred at 30°C for 48 h. The products were harvested after centrifugation at 8000 rpm for 10 min and washed multiple times with PBS. To explore the NIR-responsive release of NO, GNCs@NO (1 mg) were dispersed in phosphate-buffered saline (PBS) solution with or without exposure to 808 nm of NIR irradiation (1 W/cm^2^) for 5 min. Then, the GNCs@NO were centrifuged at 8000 rpm for 10 min, and the supernatant was collected for the NO Griess assays. Briefly, 100 μL of the supernatant was added to Griess agent I and placed in the dark for 15 min. Subsequently, Griess agent II was slowly added to the mixture and allowed to react in the dark for 15 min to produce a diazo compound. We then quantified NO by measuring the UV absorbance of the solution of the generated diazo compound at 540 nm.

### 2.3 Cytotoxicity of GNCs and GNCs@NO

The cytotoxicity of GNCs and GNCs@NO toward human breast carcinoma MCF-7 cells and human breast epithelial MCF-10A cells was evaluated in the presence or absence of NIR irradiation using an SRB assay. Briefly, MCF-7 and MCF-10A cells were respectively seeded into 96-well plates (5×10^3^ cells/well) and cultured overnight. GNCs or GNCs@NO were then added to these cells at various concentrations and co-incubated for 24 h. Next, 100 ml of 20% trichloroacetic acid was added to each well, followed by incubation at 4°C for 3 h. Then, the cell media were discarded and the cells were washed three times in PBS. Subsequently, 100 ml of 0.4% w/v SRB solution was added to each well. After 30 min, the unbound SRB was discarded, and 150 ml of 10 mM Tris-HCl was added to each well, followed by shaking for 5 min to solubilize the bound SRB. The optical density (OD) was measured at 570 nm, and the relative cell viability was calculated according to the ratio of the OD value to that of the control group. For the NIR irradiation group, GNCs and GNCs@NO (12.5 μg/ml) were respectively added to MCF-7 cells and co-cultured for 24 h and subsequently irradiated with NIR light (808 nm, 1 W/cm^2^) for 5 min. To evaluate the radiosensitization effect of GNCs and GNCs@NO, the cells were treated with saline, GNCs, or GNCs@NO (12.5 μg/ml) for 24 h and irradiated with X-rays (1 Gy/min for 5 min). Subsequently, cell viability was assessed using an SRB assay.

### 2.4 ROS and RNS measurement

The MCF-7 cells were treated with GNCs or GNCs@NO (12.5 μg/ml) for 24 h and then exposed to 808 nm NIR light (1 W/cm^2^) for 5 min or/and irradiated with 1 Gy/min of X-ray for 5 min. These MCF-7 cells were then mixed with dichlorodihydrofluorescein diacetate (DCFH-DA) and incubated for 20 min. Subsequently, the fluorescence intensity of DCFH-DA was measured by flow cytometry to measure intracellular ROS levels. To evaluate intracellular RNS levels, the cells were incubated with dihydrorhodamine (DHR) for 20 min after different treatments. The fluorescence intensity of DHR was determined by flow cytometry.

### 2.5 Colony formation assay

MCF-7 cells were seeded into 25 cm^2^ flasks and cultured for 24 h. Then, these MCF-7 cells were co-incubated with GNCs or GNCs@NO or/and treated with NIR or/and X-ray irradiation. After 24 h of administration, the cells were trypsinized and cultured in 6 cm dishes for 7 days. The colonies were stained with 3-(4,5-dimethylthiazol-2-yl)-2-5-diphenyltetrazolium bromide. Colonies containing over 50 cells were counted.

### 2.6 *In vivo* combination therapies

We purchased six-week-old female nude mice from the Animal Experimental Center of Jilin University and kept them at a conventional animal housing facility in The Second Hospital of Jilin University. All the animal experimental protocols and experimental operations were approved by the Ethics Committee for the Use of Experimental Animals of The Second Hospital of Jilin University. To establish MCF-7 xenograft models, the nude mice were anesthetized by inhaling 2% isoflurane and subsequently injected with 5 × 10^6^ MCF-7 cells into the mammary fat pads. When the tumor volume reached approximately 0.08 cm^3^, the MCF-7 xenograft models were divided into seven groups: saline, NIR, X-ray, GNCs + X-rays, GNCs + NIR, GNCs@NO, and GNCs@NO + X-rays + NIR. Mice in the nanoparticle-treated groups were intravenously injected with GNCs or GNCs@NO at a dose of 5 mg/kg every 3 days for a total of four administrations. In the NIR-exposed groups, the tumor tissues were exposed to an 808 nm of NIR light (1 W/cm^2^) for 5 min 6 h after the injection of nanoparticles or PBS. In the X-ray-irradiated groups, the tumor sites were irradiated with 1 Gy/min of X-ray for 5 min 6 h after the injection of nanoparticles or PBS. The tumor lengths and widths were measured using a digital caliper 2 days post-administration. We subsequently calculated the tumor volumes using the following equation: tumor volume = length × width^2^ × 0.52. The experiment was terminated on day 18. All the mice were euthanized by carbon dioxide asphyxia and the tumors were collected for weighing. Serum was collected to assess biochemical parameters. The major organs, including the liver, spleen, kidney, lung, and heart, were harvested, fixed, and stained with hematoxylin and eosin.

### 2.7 Statistical analysis

The differences between two groups were analyzed using Student’s t-tests. Differences between more than two groups were analyzed using one-way analysis of variance. Statistical significance was set at *p* < 0.05.

## 3 Results and discussion

**FIGURE 1:**
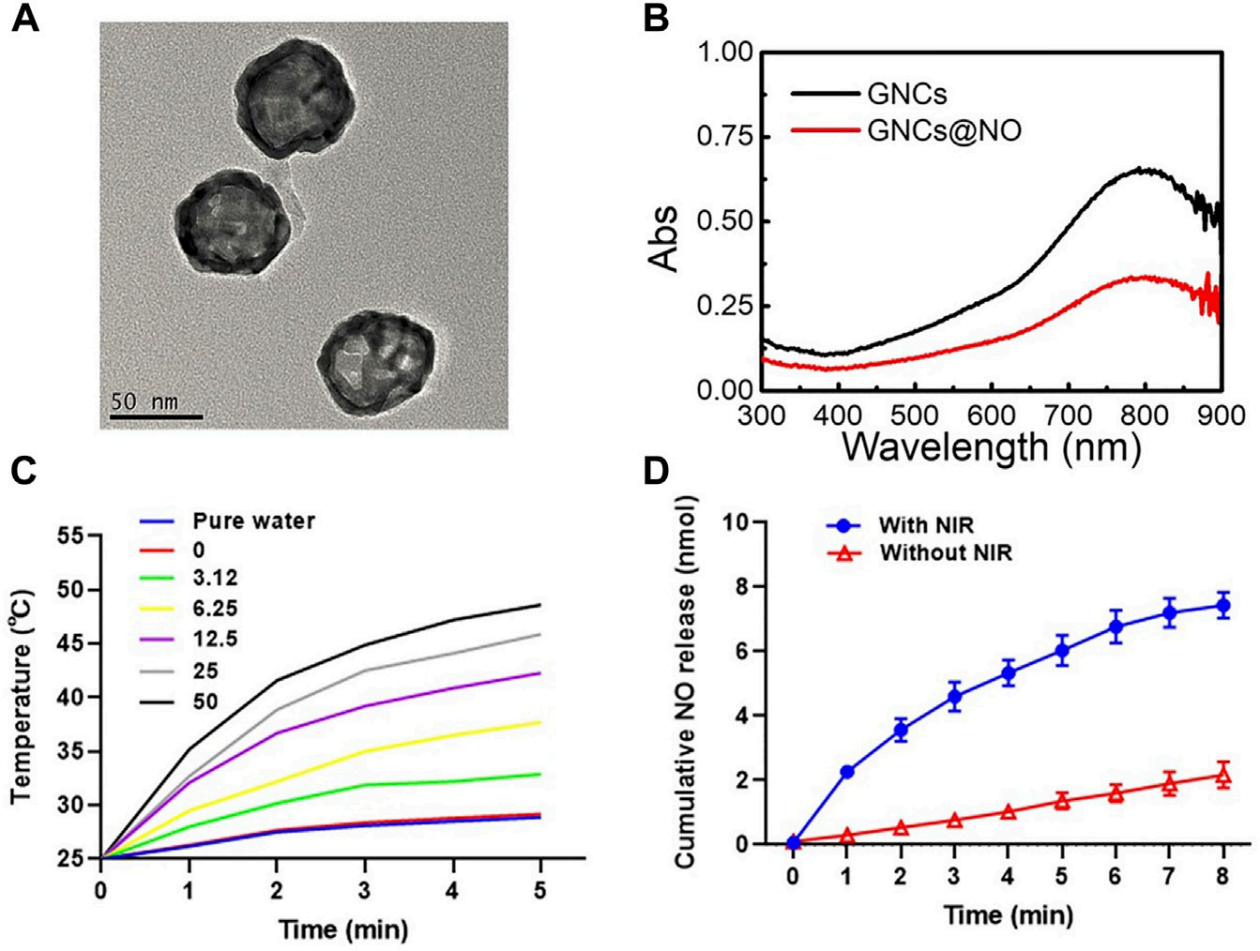
Characterization of GNCs@NO. **(A)** TEM image of GNCs. **(B)** UV-vis spectra of GNCs and GNCs@NO. **(C)** Temperature change curve of GNCs@NO suspensions with exposure to NIR irradiation. **(D)** Cumulative release of NO gas from GNCs@NO according to exposure to NIR irradiation.

GNCs were fabricated *via* a galvanic replacement reaction between HAuCl_4_ and silver nanocages as previously reported (Tang et al., 2021). Transmission electron microscopy (TEM) images indicated the uniform morphology and good monodispersity of the prepared GNCs (Figure 1A). The dynamic light scattering assay showed an average GNC size of approximately 110.4 nm (Supplementary Figure S1), with a polydispersity index of 0.184. Additionally, the GNCs showed hollow structures and porous walls (Supplementary Figure S2). Subsequently, the surface of the prepared GNCs was modified with thiolate cupferron through thiol–gold interactions. The hydrodynamic size of the GNCs@NO was about 130.3 nm with a PDI value of 0.15 (Supplementary Figure S3) and a GNCs@NO surface zeta-potential of −12.2 mV, which was higher than that of the GNCs (−15.4 mV) (Supplementary Figure S4). Furthermore, the localized surface plasmon resonance peak shifted from 792 to 800 nm (Figure 1B), suggesting the successful modification of thiolate cupferron. Moreover, the strong absorption of GNCs@NO in the NIR region implied that GNCs@NO had good photothermal conversion ability. To explore the photothermal conversion effect, cell culture media dispersions of GNCs@NO at different concentrations were irradiated by an 808 nm laser at a power density of 1 mW/cm^2^. As shown in Figure 1C, after 5 min of continuous NIR irradiation, the changes in temperature in the water or cell culture medium were <6°C, whereas the GNCs@NO samples showed time- and concentration-dependent temperature increases. After irradiation with NIR light for 5 min, the temperature of the 12.5 μg/ml GNCs@NO sample was >43.2°C, a crucial temperature to induce tumor cell death without normal cell death. We then investigated the release of NO from GNCs@NO in the absence or presence of NIR irradiation. After NIR irradiation, GNCs@NO (12.5 μg/ml) showed a temperature change like that of GNCs at the same concentration (Supplementary Figure S5), indicating that the NO donor loading had a negligible effect on the photothermal conversion of GNCs. As illustrated in Figure 1D, NIR irradiation remarkably accelerated the release of NO from GNCs@NO, which was attributed to the slow decomposition of thiolate cupferron as an N-hydroxy-N-nitrosamine NO donor and release of NO under physiological conditions and rapid decomposition with the generation of NO at high temperature (Wang et al., 2002). The NIR-responsive NO release of GNCs@NO further confirmed their excellent photothermal conversion efficiency. To improve the biostability, GNCs@NO were functionalized with polyethylene glycol (PEG). GNCs@NO showed long-term stability in the cell culture media after PEGylation, whereas GNCs@NO without PEG modification showed an aggregate (Supplementary Figure S6).

**FIGURE 2:**
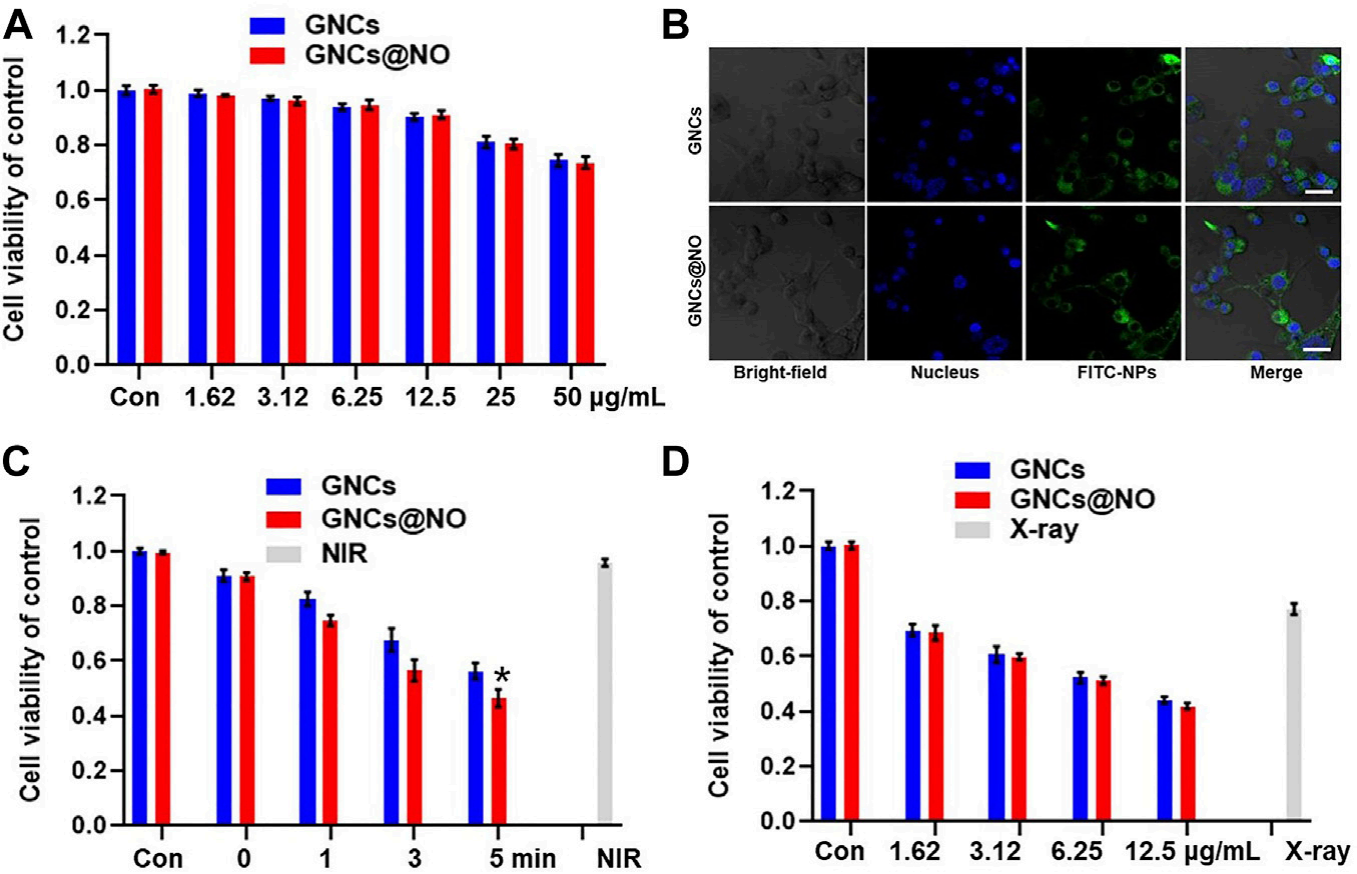
Photothermal therapy and radiosensitization of GNCs@NO. **(A)** Cytotoxicity of GNCs and GNCs@NO at various concentrations in MCF-7 cells. **(B)** CLSM images of MCF-7 cells co-incubated with GNCs or GNCs@NO for 3 h. Scale bar = 10 μm. **(C)** Viability of MCF-7 cells after incubation with GNCs or GNCs@NO with NIR irradiation. *p < 0.05 *versus* GNC group. **(D)** Viability of MCF-7 cells after incubation with GNCs or GNCs@NO with exposure to X-ray irradiation. The values represent mean values ±SD, *n* = 5.

Biosafety is particularly important for the biomedical application of nanoparticles (Yang et al., 2013; Xia et al., 2014; Kozics et al., 2021). Therefore, we investigated the cytotoxicity of various concentrations of GNCs and GNCs@NO toward MCF-7 and MCF-10A cells. As illustrated in Figure 2A and Supplementary Figure S7, GNCs and GNCs@NO both exhibited concentration-dependent cytotoxicity against the two cell lines. Additionally, the cytotoxicity of GNCs@NO was similar to that of GNCs, possibly because of the weak release of NO under physiological conditions. The viabilities of MCF-7 and MCF-10A cells were both >90% at GNC concentrations <12.5 μg/ml. Considering its lower cytotoxicity, a concentration of 12.5 μg/ml was chosen as the optimum dose in subsequent therapies. Additionally, both GNCs@NO and GNCs showed high cellular internalization efficiency in MCF-7 cells, indicating their good biocompatibility (Figure 2B and Supplementary Figure S8). Subsequently, we investigated the PTT effects of GNCs and GNCs@NO *in vitro*. Compared to the control groups, the viability of MCF-7 cells was <5% even 5 min after NIR irradiation (Figure 2C), confirming the biosafety of the applied NIR. In contrast, NIR irradiation induced time-dependent cytotoxicity toward MCF-7 cells after treatment with GNCs or GNCs@NO, indicating that our prepared nanoparticles could be used as effective photothermal agents for PTT. Notably, GNCs@NO showed a stronger killing effect compared to GNCs owing to NO-poisoning induced by the released NO gas. We subsequently explored the radiosensitizing effects of GNCs and GNCs@NO. MCF-7 cells irradiated with X-rays without nanoparticle treatment showed a high cell count (Figure 2D). In contrast, the viability of MCF-7 cells was reduced after treatment with nanoparticles combined with X-ray irradiation. Moreover, the killing effect of X-rays increased with increasing concentration of GNCs or GNCs@NO, indicating the radiosensitizing effect of GNCs and GNCs@NO.

**FIGURE 3:**
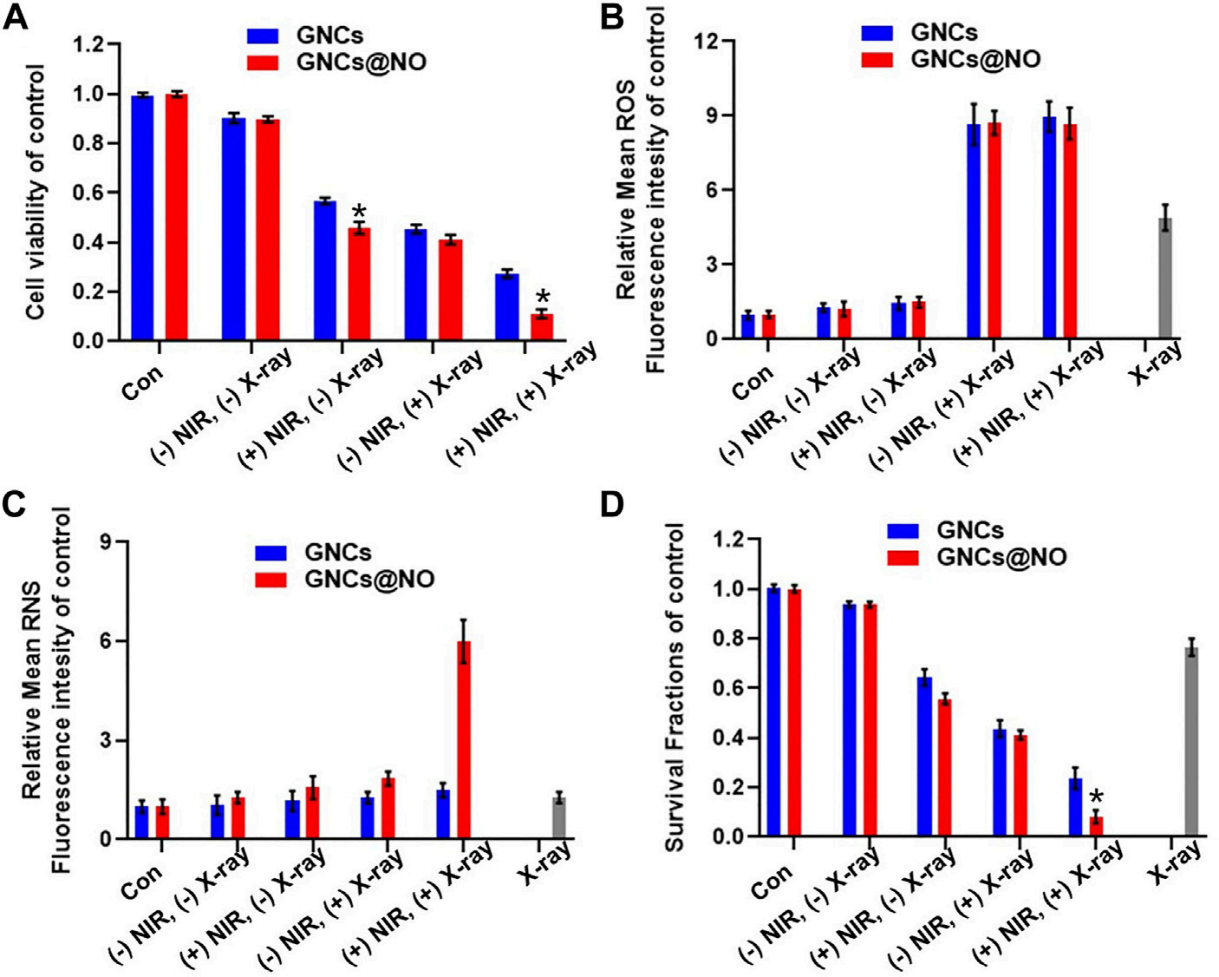
Combined GNCs@NO therapies *in vitro*. **(A)** Viability of MCF-7 cells after incubation with GNCs or GNCs@NO with (+) or without (-) NIR or/and X-ray irradiation, *p < 0.05 *versus* GNC group. **(B)** Quantitative analysis of ROS production by FACS. **(C)** Quantitative analysis of RNS production by FACS. **(D)** Colony formation assay of MCF-7 cells for various treatments, *p < 0.05 *versus* GNC group. The values represent mean values ±SD, *n* = 5.

After demonstrating the PTT effect and NIR-responsive NO gas therapy, as well as the radiosensitizing effect of GNCs@NO, we explored the combined therapeutic efficacy of GNCs and GNCs@NO with exposure to X-rays and NIR light for 5 min *in vitro*. As shown in Figure 3A, nanoparticles combined with irradiation with both NIR light and X-rays destroyed significantly more MCF-7 cells compared to the combination with only NIR or X-ray irradiation, suggesting a good combination efficiency of radio-photothermal treatments. More importantly, GNCs@NO + X-rays + NIR treatment exhibited higher cytotoxicity toward MCF-7 cells than GNCs + X-rays + NIR. To explore the underlying mechanism, we measured the levels of intracellular ROS and RNS after various treatments. As shown in Figure 3B, neither GNCs@NO nor GNCs induced detectable ROS or RNS production with or without NIR irradiation. Additionally, X-ray irradiation led to the generation of ROS in MCF-cells, while both GNCs@NO and GNCs improved the ROS generation induced by X-ray irradiation, further confirming the radiosensitizing efficacy of GNCs and GNCs@NO. More importantly, a significant enhancement in RNS signal was observed in the GNCs@NO + X-rays + NIR treatment group, whereas few RNS were detected in the GNCs + X-rays + NIR group (Figure 3C). The generation of more toxic RNS originates from the reaction of ROS with the released NO, suggesting the synergistic effects of NO therapy with radiotherapy. To further explore the long-term therapeutic efficacy of GNCs@NO, we investigated the clonal ability of MCF-7 cells after different treatments using a colony formation assay. As illustrated in Figure 3D, the number of MCF-7 colonies decreased after X-ray irradiation, whereas a smaller surviving fraction was observed in the GNCs + X-rays group owing to the radiosensitizing effect of GNCs. GNCs + X-ray + NIR exhibited a stronger inhibition of colony formation of MCF-7 cells compared to GNCs + X-rays owing to the combined effect of PTT. Consistent with the cell viability results, the smallest number of MCF-7 colonies was observed in the GNCs@NO + X-rays + NIR treatment group. These results confirmed that GNCs@NO, which integrates PTT, radiosensitization, and NIR-responsive NO gas therapy, were an effective combined therapy against TNBC.

**FIGURE 4:**
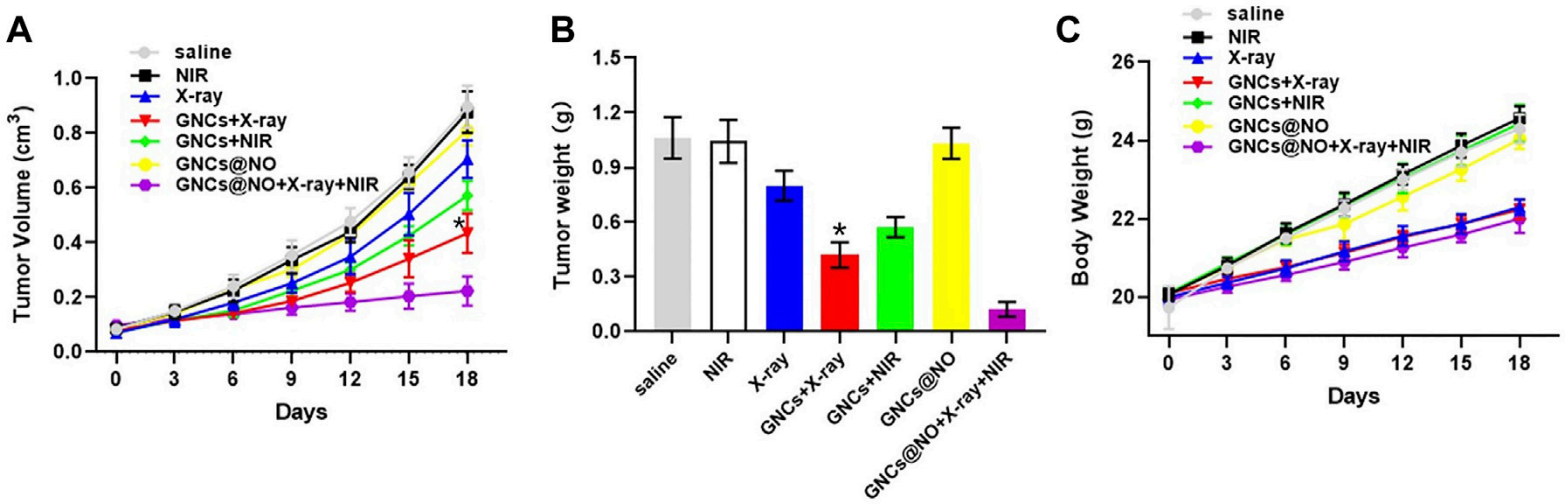
*In vivo* GNCs@NO combination therapies. **(A)** Tumor growth curve, *p < 0.05 *versus* X-ray group. **(B)** Tumor weight, *p < 0.05 *versus* X-ray group. **(C)** Body weight. The values represent mean values ±SD, *n* = 5.

Encouraged by the *in vitro* efficacy of PTT/radiotherapy/gas therapy, we explored the ability of the GNCs@NO + X-rays + NIR treatment to delay tumor progression *in vivo*. MCF-7 xenografts were replicated and divided into seven groups: saline, NIR, X-ray, GNCs + X-rays, GNCs + NIR, GNCs@NO, and GNCs@NO + X-rays + NIR; the treatments were administered by tail vein injection of drugs and/or X-ray irradiation in the absence or presence of NIR every 3 days for a total of six administrations. As illustrated in Figures 4A, B, tumors in mice treated with saline or NIR grew rapidly, whereas GNC + NIR treatment showed significant tumor growth inhibition compared to the NIR and control groups, indicating the PTT efficacy of GNCs. Additionally, GNCs@NO had a negligible inhibitory effect on tumor growth because of the weak release of NO gas. The X-ray group also showed an unsatisfactory inhibitory effect due to the radioresistance of the tumor tissues. However, the inhibitory effect of X-rays was improved in the GNCs + X-rays treatment group, further confirming the radiosensitization effect of GNCs. GNCs@NO + X-rays + NIR induced nearly complete inhibition of tumor growth. These findings demonstrate that GNCs@NO had excellent antitumor efficacy through the synergistic effects of PTT, radiosensitization, and gas therapy.

**FIGURE 5:**
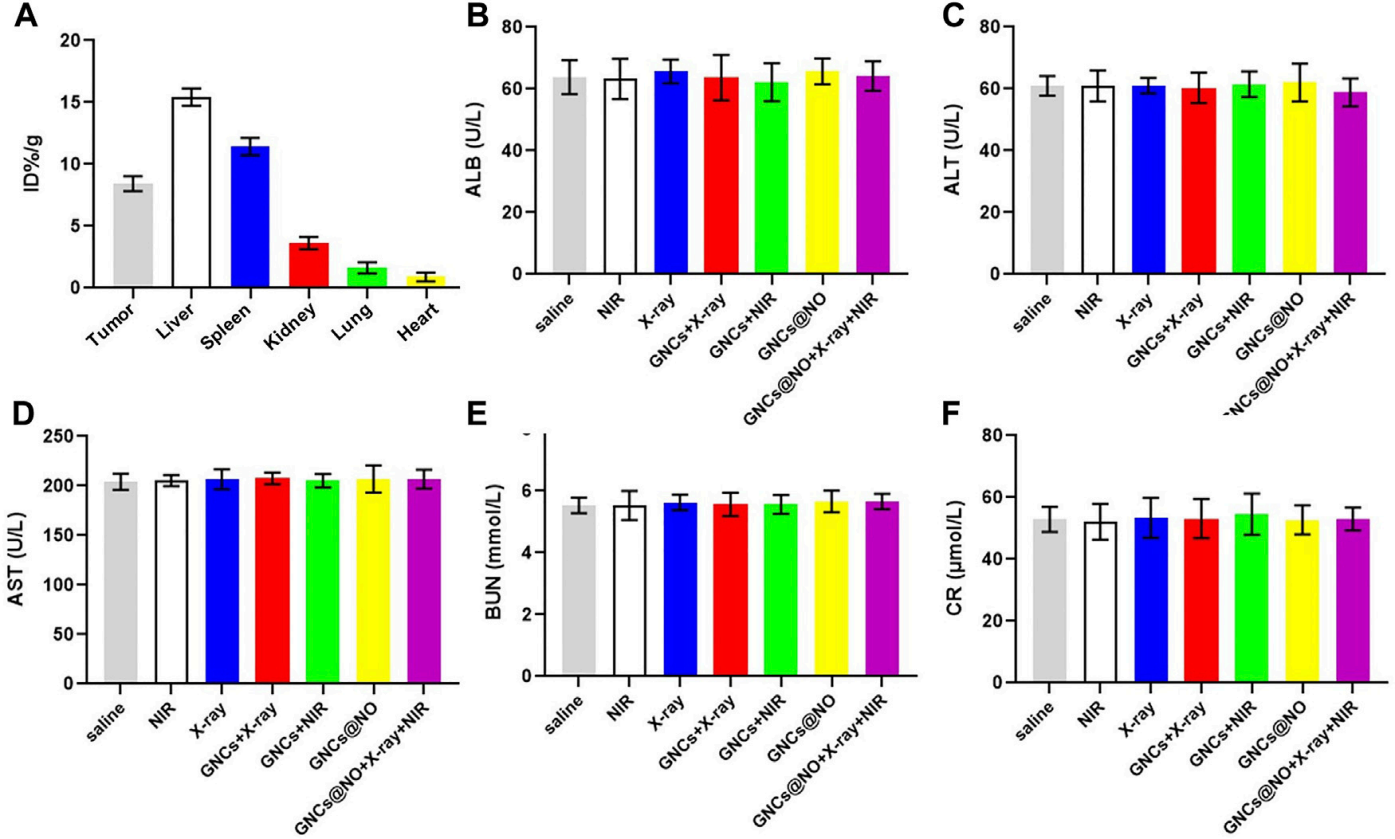
Biosafety evaluation. **(A)** Biodistribution of GNCs@NO. **(B–F)** Serum biochemistry indexes. Levels of ALB **(B)**, ALT **(C)**, AST **(D)**, BUN **(E)**, and CRE **(F)**. The values represent mean values ±SD, *n* = 3.

**FIGURE 6:**
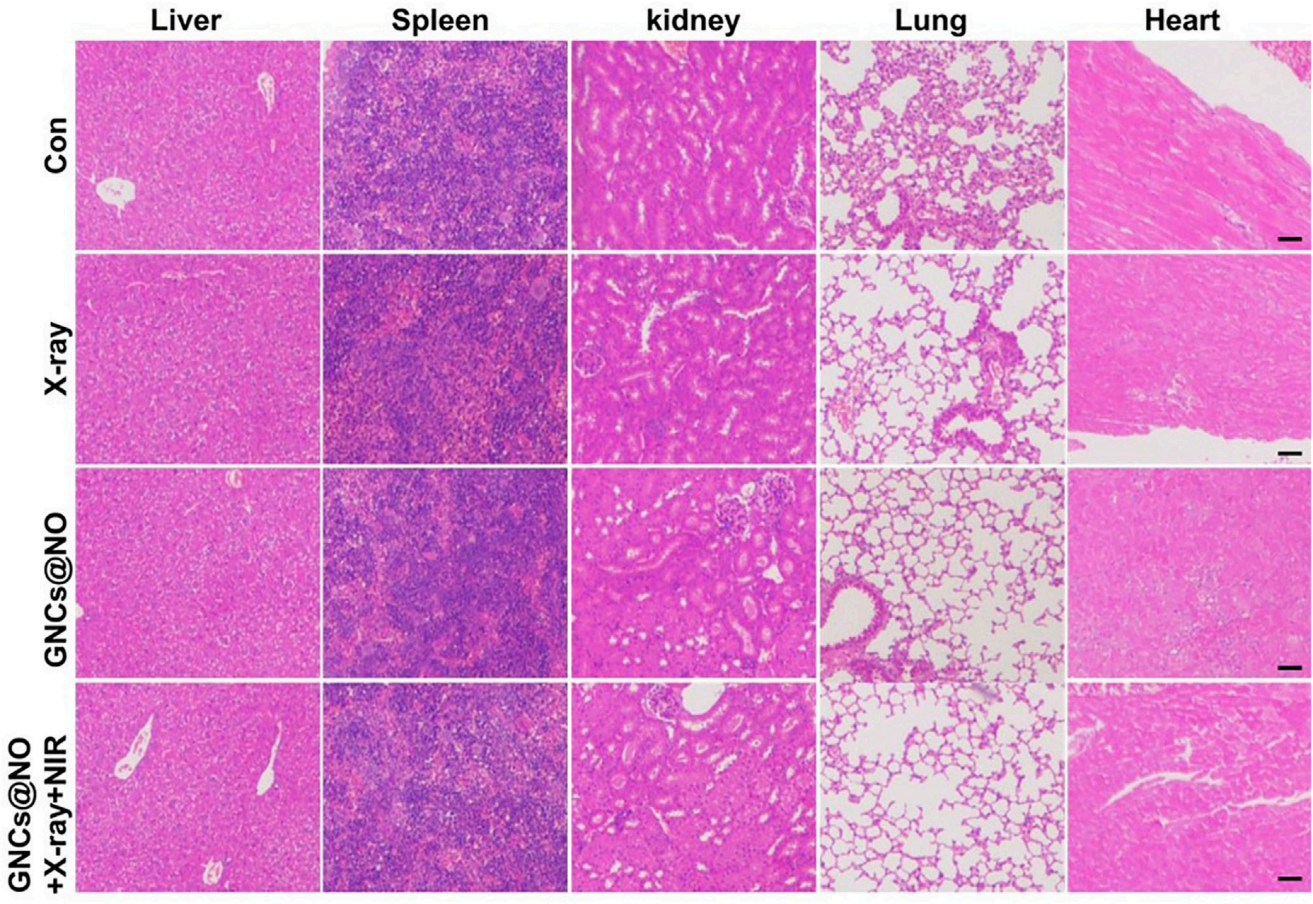
Histopathology of the livers, spleens, kidneys, lungs, and hearts from MCF-7 tumor-bearing mice from each group. Scale bar = 50 μm.

After confirming the outstanding anticancer efficiency, we evaluated the accumulation of GNCs@NO in tumors and major organs, including the heart, liver, spleen, lungs, and kidneys. As shown in Figure 5A, GNCs@NO predominantly accumulated in the reticuloendothelial system of the tumor tissues, liver, and spleen. Furthermore, the body weight of mice after treatment with X-rays decreased due to radiotherapy-induced gastrointestinal injury (Li et al., 2010; Li et al., 2019; Xie et al., 2020). In contrast, GNCs@NO did not induce a change in body weight compared to the saline group, while the body weight of the mice treated with GNCs + X-rays or GNCs@NO + X-rays + NIR showed no observable difference from the body weight of mice treated with X-rays (Figure 4C). These results suggested that GNCs@NO-based combination therapy might not aggravate radiotherapy-induced side effects. More importantly, blood biochemistry indices, including alkaline phosphatase (ALB), aspartate aminotransferase (AST), alanine aminotransferase (ALT), blood urea nitrogen (BUN), and creatinine (CR) (Figures 5B, F), do not exhibit significant changes in any of the treatment groups compared to the saline group, indicating good liver and kidney functions during the combined treatment. Furthermore, no pathological changes were observed in the major organs of the mice after treatment with GNCs@NO + X-rays + NIR (Figure 6). Overall, these findings confirmed the low systemic toxicity of GNCs@NO-based combination therapies.

## 4 Conclusion

We designed a hyperpyrexia-sensitive NO donor (GNCs@NO) to integrate PTT, radiosensitization, and NIR-controlled NO gas therapy. GNCs@NO showed high drug loading of the NO donor and high photothermal conversion efficiency, which not only generated hyperpyrexia to induce cancer cell death but also facilitated rapid NO release under NIR irradiation. Additionally, we confirmed the radiosensitizing efficacy and NIR-activated NO gas therapy of GNCs@NO *in vitro* and *in vivo*. More importantly, GNCs@NO + X-ray + NIR treatment induced more lethal RNS production through the reaction of the released NO with the ROS induced by radiotherapy. Therefore, GNCs@NO + X-rays + NIR exerted outstanding anticancer efficacy due to synergistic effects. Furthermore, the body weight measurement, blood biochemistry analysis, and organ histopathology staining indicated the low systemic toxicity of GNCs@NO-based combination treatments. Consequently, GNCs@NO are a promising nanoplatform for combined radio/photothermal/gas therapy for TNBC.

## Data Availability

The original contributions presented in the study are included in the article/Supplementary Material. Further inquiries can be directed to the corresponding author.

## Supplementary material

Supplementary Figure S1 Hydrodynamic size of GNCs.

Supplementary Figure S2 HRTEM of GNCs.

Supplementary Figure S3 Hydrodynamic size of GNCs@NO.

Supplementary Figure S4 Surface zeta potential of GNCs and GNCs@NO.

Supplementary Figure S5 Temperature changes in GNC suspensions at 12.5 μg/mL with exposure to NIR irradiation.

Supplementary Figure S6 Long-term stability of GNCs@NO with or without PEG modification in cell media.

Supplementary Figure S7 Cytotoxicity of GNCs and GNCs@NO against MCF-10A at various concentrations.

Supplementary Figure S8 Quantitative analysis of the cellular internalization of GNCs and GNCs@NO in MCF-7 cells.
