# Supplementary material for: Gold nanoplatform for near-infrared light-activated radio-photothermal gas therapy in breast cancer
Source: Front Bioeng Biotechnol. 2023 Jan 6;10:1098986. doi: 10.3389/fbioe.2022.1098986 (PMC9853036; doi:10.3389/fbioe.2022.1098986)
Supplement: Supplementary file 1 [file Presentation1.pdf]

Supplementary Material

Gold Nanoplatforms for Near-Infrared Light-Activated Radio-Photothermal-Gas Therapy of Breast Cancer

Shuting Zuo [1], Zhenyu Wang [1], Liping Zhao [2], Jing Wang[1,] *

[1]Department of Breast Surgery, The Second Hospital of Jilin University, Changchun, 130041, PR China

[2]Gynecology and Obstetrics Department of the Second Hospital of Jilin University, Changchun, 130041, PR China

*** Correspondence:**
Corresponding Author
wangjing001@jlu.edu.cn

1 Materials

Gold chloride hydrate (HAuCl4), silver trifluoroacetate, sodium hydrosulfide hydrate, thioacetic acid, Poly(vinylpyrrolidone) (PVP) and dimethyl sulfoxide-d6 (DMSO-d6) were purchased from the Sigma-Aldrich Co. (St Louis, MO, USA). RPMI-1640 medium and fetal bovine serum (FBS) were obtained from GIBCO. All of the reagents and can be used without further purification.

2 Animal model establishment

Six-week-old female nude BALB/c nude mice were purchased from the Animal Experimental Center of Jilin University. These mice were maintained in regular cages in a traditional animal house at the Second Hospital of Jilin University. All animal experimental protocols were approved by the Ethics Committee for the Use of Experimental Animals of the Second Hospital of Jilin University. All the operations met a criterion of the National Institute of Health Guide for the Care and Use of Laboratory Animals. To establish MCF-7 tumor -bearing mouse models, 5 × 106 MCF-7 were injected into the into the mammary fat pads of these mice. When the volume of tumors reached about 80 mm3, the mice were administrated with various treatments.

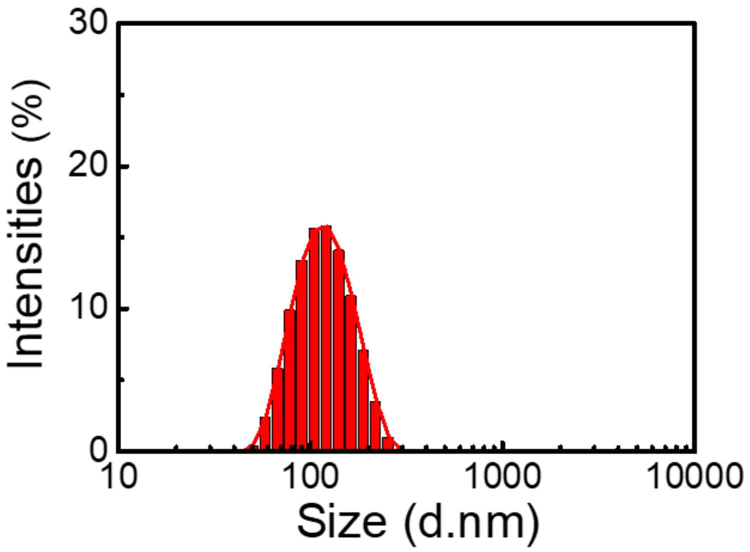

Supplementary Figure 1. Hydrodynamic size of GNCs.

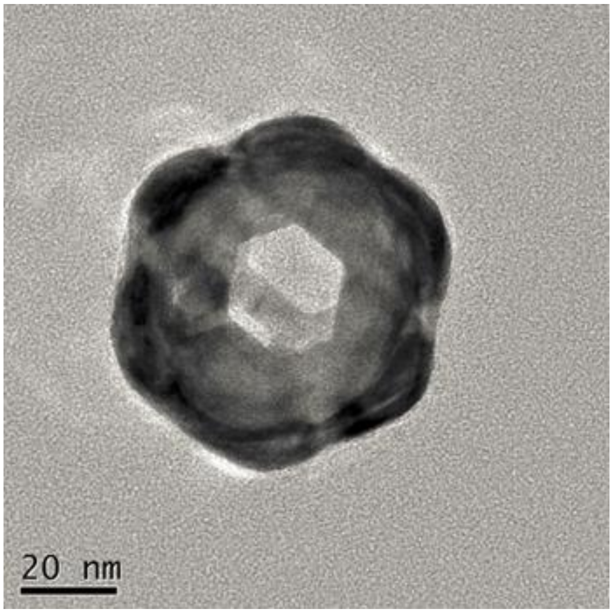

Supplementary Figure 2. HRTEM of GNCs.

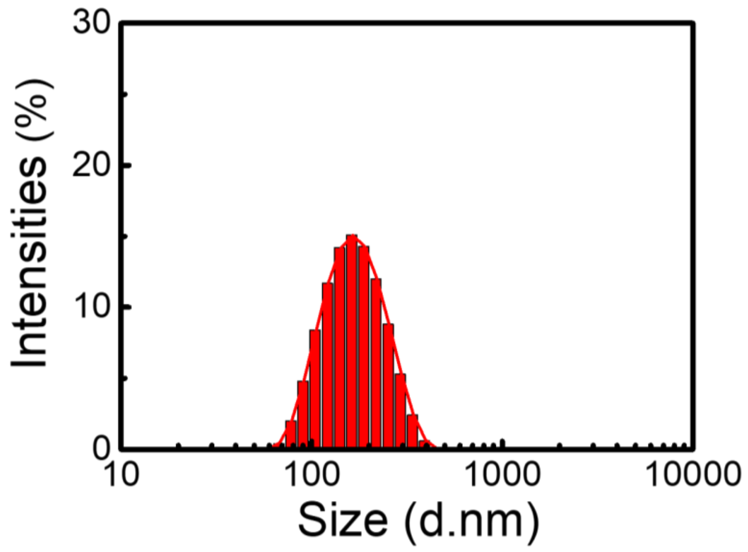

Supplementary Figure 3. Hydrodynamic size of GNCs@NO.

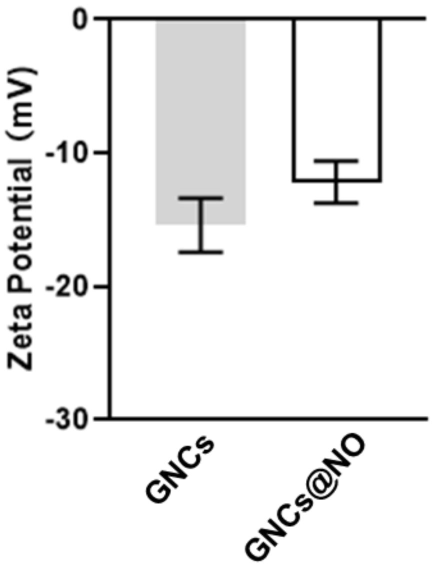

Supplementary Figure 4. Surface zeta potential of GNCs and GNCs@NO.

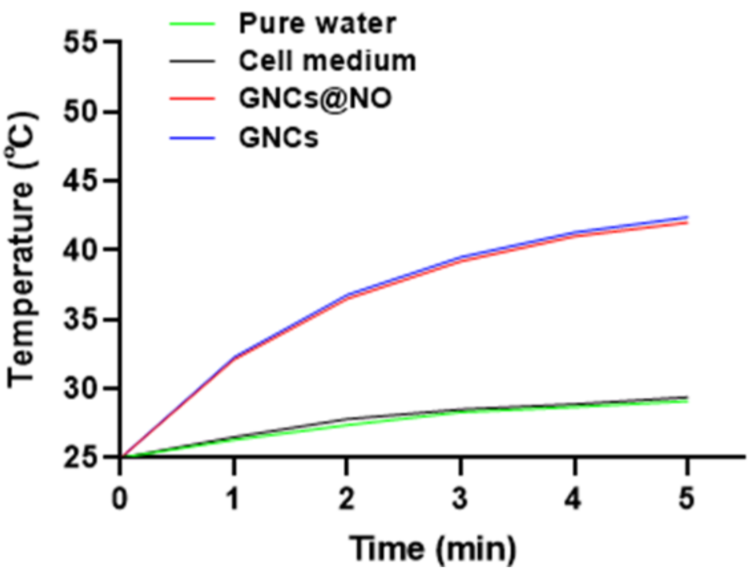

Supplementary Figure 5. Temperate change of GNCs suspensions at a concentration of 12.5 μg/mL with an exposure of NIR irradiation.

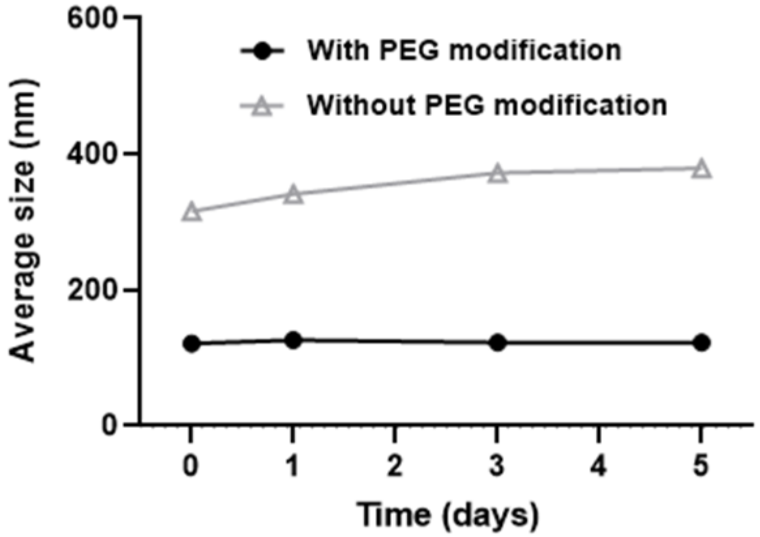

Supplementary Figure 6. Long-term stability of GNCs@NO with or without PEG modification in cell medium.

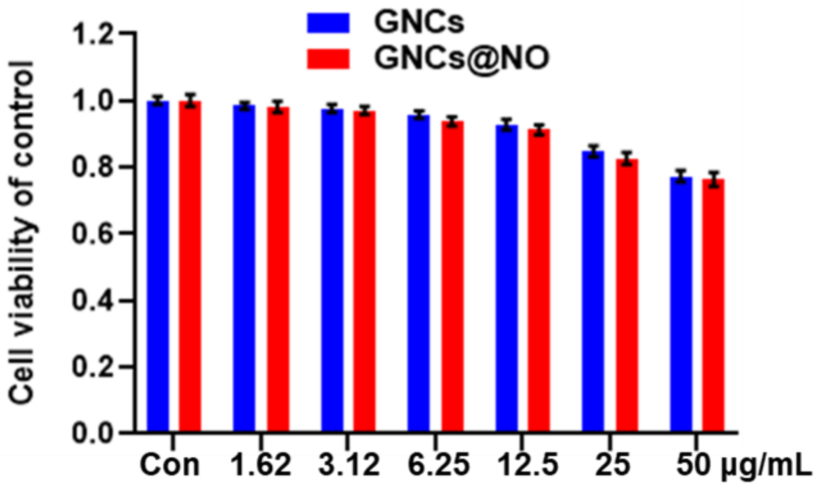

Supplementary Figure 7. Cytotoxicity of GNCs and GNCs@NO against MCF 10A at various concentrations.

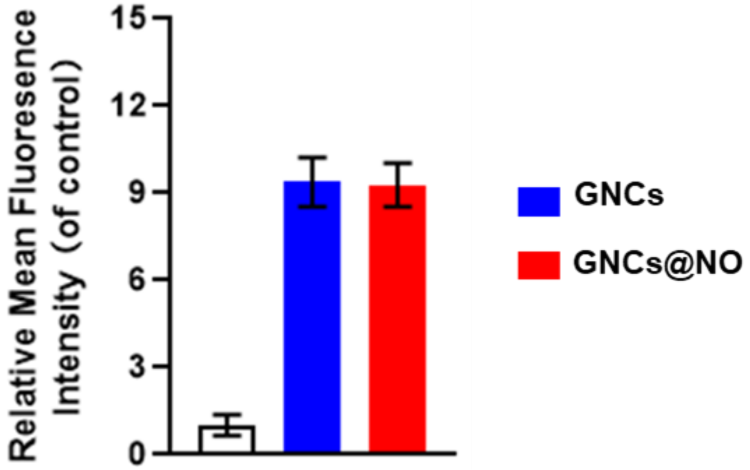

Supplementary Figure 8. Quantitative analysis of the cellular internalization of GNCs and GNCs@NO with MCF-7 cells.
